# A High Performance Piezoelectric Sensor for Dynamic Force Monitoring of Landslide

**DOI:** 10.3390/s17020394

**Published:** 2017-02-17

**Authors:** Ming Li, Wei Cheng, Jiangpan Chen, Ruili Xie, Xiongfei Li

**Affiliations:** Institute of Solid Mechanics, Beihang University, Beijing 100191, China; chenjiangpan@126.com (J.C.); xieruili890712@126.com (R.X.); xiongfei_li0908@163.com (X.L.)

**Keywords:** piezoelectric sensor, calibration experiment, dynamic measurement, landslides, landslide monitoring and forecasting system, low-frequency correction

## Abstract

Due to the increasing influence of human engineering activities, it is important to monitor the transient disturbance during the evolution process of landslide. For this purpose, a high-performance piezoelectric sensor is presented in this paper. To adapt the high static and dynamic stress environment in slope engineering, two key techniques, namely, the self-structure pressure distribution method (SSPDM) and the capacitive circuit voltage distribution method (CCVDM) are employed in the design of the sensor. The SSPDM can greatly improve the compressive capacity and the CCVDM can quantitatively decrease the high direct response voltage. Then, the calibration experiments are conducted via the independently invented static and transient mechanism since the conventional testing machines cannot match the calibration requirements. The sensitivity coefficient is obtained and the results reveal that the sensor has the characteristics of high compressive capacity, stable sensitivities under different static preload levels and wide-range dynamic measuring linearity. Finally, to reduce the measuring error caused by charge leakage of the piezoelectric element, a low-frequency correction method is proposed and experimental verified. Therefore, with the satisfactory static and dynamic properties and the improving low-frequency measuring reliability, the sensor can complement dynamic monitoring capability of the existing landslide monitoring and forecasting system.

## 1. Introduction

As a type of the most severe geological disasters, landslides can cause economic losses for billions of dollars around the world annually. Roads, bridges, oil-gas pipelines, and other infrastructures in mountain regions, as well as the people’s lives and property, are seriously threatened by landslides [1].

Establishing the landslide monitoring and forecasting system (LMFS) is a feasible way to degrade the damage. Based on the manual methods, the early LMFSs [2,3] are built by observing the variation of the displacement of ground surface, the groundwater level, the plants and the other parameters in the slope area. With the development of science and technology, some mechanical instruments such as theodolites, inclinometers, and level gauges are used in the LMFSs [4,5]. However, due to the limited accuracy of the traditional measuring instruments, the early LMFSs can hardly satisfy the increasing engineering requirements.

Recently, by using the modern measuring instruments and techniques, several significant achievements in slope engineering fields have been obtained. Based on GPS and geodetic techniques, Puglisi et al. [6] developed a remote system of ground deformation monitoring, which can real-timely measure the slope displacement and transmit signals wirelessly. Zhang et al. [7] established a real-time remote system to monitor the landslides around the highway in mountain regions by utilizing the general packet radio service (GPRS) net of China Telecom. Aimed at the slopes of mountain highways, a remotely controlled system was built by Wu et al. [8] to monitor and forecast the disasters by using GPRS, the trigger displacement meter, the grid pluviometer, and the other advanced techniques. Interferometry synthetic aperture radar (InSAR) was used by Perski et al. [9] to measure the terrain deformation near the Wieliczka Salt Mine in Poland. Jia et al. [10] proposed a static and dynamic factors-coupled forecasting model of regional rainfall-induced landslides, which quantitatively considered both the static and dynamic factors including the geological and geographical factors.

Although the modern methods and techniques are truly helpful to accurately measure the change of the parameters including the slope deformation, precipitation, soil moisture content and even the seepage pressure, the LMFSs still can hardly forecast the landslide efficiently as expected. Then, the geologists denoted that the variation of the above parameters is a necessary, but not sufficient, condition to the occurrence of the landslide.

As is well known, according to the Newton’s First Law, force is the source of the change of motion state. The landslide, as a kind of “motion”, is closely related to the change of the “force” inside the slope. Therefore, the sliding force in the potential landslide area should be regarded as the core parameter to be effectively measured. Accordingly, the force sensors are the key components in an effective LMFS.

Each type of sensor has its own advantages and limitations during the application. Due to the different properties of the key force-sensing element, the common force sensors can be divided into static and dynamic types.

In the aspect of static measurement, for instance, the differential resistor sensor [11] and the resistance strain-gauge sensor [12] are normally used into general industrial projects and laboratory applications. Vibrating wire sensors [13,14] are utilized in high stress fields for quasi-static tests due to the properties of high compressive strength. Wang et al. [15] designed an improved type of vibrating wire sensor to measure the anchorage stress in underground engineering.

In the aspect of dynamic measurement, piezoelectric sensors are frequently used. By using piezoelectric acoustic emission sensors, Agioutantis et al. [16] monitored the failure process of the Nestos marble in three points bending tests, and investigated the potential for accurate prediction of rock damage based on the measuring results. Karayannis et al. [17] used the embedded cement-based piezoelectric sensors to ensure the safety of the concrete structures by measuring the dynamic force. By using the state-space method, Yan et al. [18] studied the time-dependent behaviour of a simply-supported functionally graded beam bonded with piezoelectric sensors and actuators. Yang et al. [19,20] used the dynamic sensors for damage identification in the structural health monitoring field. Gu et al. [21], Chalioris et al. [22] and Voutetaki et al. [23] used piezoelectric transducers as smart aggregates to evaluate and monitor structural health of reinforced concrete, since the piezoelectric transducers have the advantages of multiple monitoring functions such as dynamic seismic response detection, structural health monitoring and white noise response detection.

**Figure 1:**
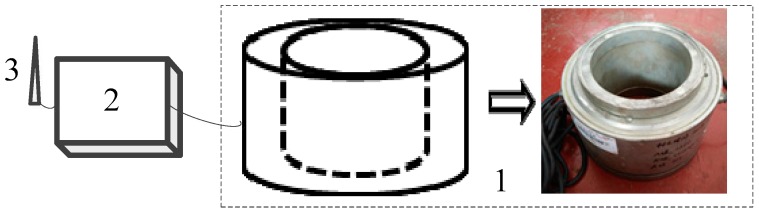
The sensing system in References [24,25,26,27,28]. (**1**)-The vibrating wire sensor; (**2**)-The signal processing system; (**3**)-The wirelessly transmitting system.

It is worth noting that He et al. [24,25,26], Tao et al. [27] and Yang et al. [28] developed a real-time remote LMFS based on the vibrating wire sensor shown in Figure 1 due to its high strength property, which has been applied in some slope engineering projects and obtained remarkable achievements by long-termly measuring the changing tension of the monitoring anchor cable. Thus, it proved that the LMFS [24,25,26,27,28] based on the force sensors has made a great breakthrough in the landslide monitoring field. However, for the reason that the working principle of the LMFS [24,25,26,27,28] depends on the long-term evolutionary trend analysis of the sliding force since the only vibrating wire sensor in the existing sensing system has the weak ability for dynamic measurement, the monitoring and forecasting efficiency of the LMFS [24,25,26,27,28] is constrained and the transient disturbing signals can hardly be captured. Unfortunately, the escaping transient disturbance has become progressively more critical to the development of landslides.

**Figure 2:**
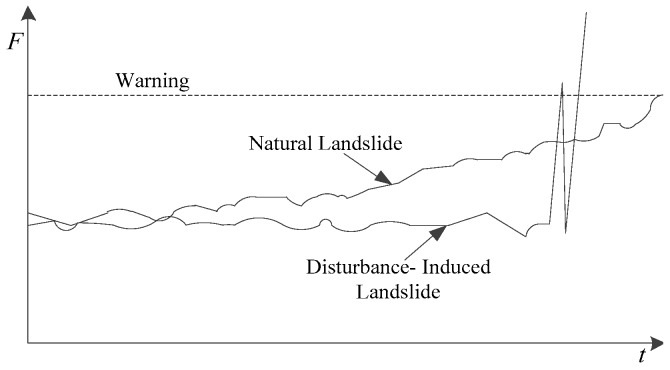
Evolution law of the sliding force for the natural landslide and disturbance-induced landslide.

On condition that the sliding force is considered as the generating mechanism, the landslides can be divided into natural and disturbance-induced types, whose evolutionary processes of sliding force are shown in Figure 2.

The natural landslide is a kind of quasi-static evolutionary behaviour. Strong weather variations, including heavy storms and blizzards, would affect the internal structure and external loading conditions in the potential landslide area. Thus, the rise of the sliding force develops together with the decline of the sliding resistance force in the slope. Once the sliding force exceeds the resistance, the landslide will occur theoretically.

The disturbance-induced landslide has a kind of relatively rapid evolutionary process. Under the natural conditions, the sliding force and the resistance are in equilibrium. The excavation unloading effect induced by human engineering activities, as well as the change of natural conditions, would make the equilibrium state vulnerable. Once an accident occurs, the consequent disturbance will abruptly break the equilibrium and cause landslides.

Currently, disturbance-induced landslides have become more frequent with the increasing human engineering activities. The randomness and non-controllability of disturbance-landslides further increase the monitoring and forecasting difficulties. Thus, the requirement of increasing the dynamic monitoring ability of the existing LMFS is more urgent.

In view of the situation that the disturbance-induced landslides occur more frequently and the lack of dynamic monitoring sensors in the existing LMFSs, as well as considering the conventional dynamic monitoring sensors can hardly adapt the high stress condition in slope engineering, a high-performance piezoelectric force sensor is presented in this paper.

This paper is organized as follows. Firstly, the piezoelectric force sensor is designed, in which two technical indexes based on the loading conditions in the practical slope engineering are prearranged and two key techniques are employed. Secondly, the advisable-dimensional prototype of the sensor is assembled, which can be theoretically validated to satisfy the two presented indexes. Thirdly, the calibration experiments are employed via the independently invented static and transient loading mechanism and the results show that the sensor has fine linearity and stability. Fourthly, the low-frequency correction method is proposed and experimental verified to improve the low-frequency measuring reliability of the sensor. Finally, the conclusions summarize the paper and state that the piezoelectric sensor can complement the existing LMFS for dynamic disturbance monitoring with its excellent static and dynamic properties.

## 2. Design of the Sensor 

**Figure 3:**
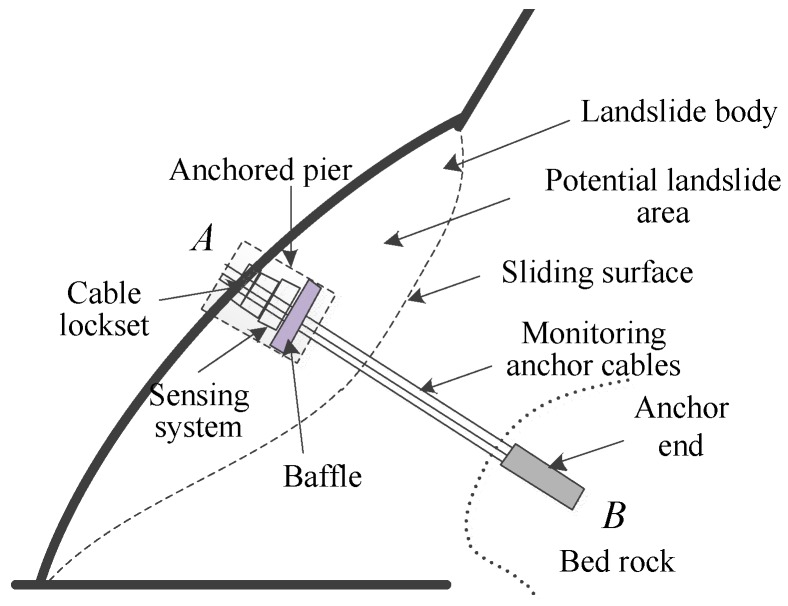
Schematic of the sliding force measuring system in slope engineering.

Figure 3 shows the schematic of the sliding force measuring system in slope engineering. As can be seen, to effectively monitor the changing forces in the monitoring anchor cable, that is, the sliding forces of the landslide body, End *B* should be anchored into the bed rock, which is a relatively stable structure inside the slope and located beyond the potential landslide area. At End *A*, an anchored pier is built, in which the sensing system are installed between the baffle and the cable locket.

### 2.1. Technical Indexes

Considering the magnitude of the sliding force and the working stress level of the anchor cables in the slope engineering projects [24,25,26,27,28], the sensor to be designed in this paper should satisfy the two technical indexes as follows.

Intex 1: The static ultimate compressive bearing capacity should reach up to 1500 kN. 

In the sensing system, both the static sensor and the dynamic sensor are installed in series and bearing the same load conditions from the monitoring anchor cable. Only with the similar high compressive strength can the sensing system play its biggest role when facing the high and complex static and dynamic stress environment in slope engineering. According to the measuring range of the vibrating wire sensor applied in the existing LMFS shown in Figure 1, we set 1500 kN as the static ultimate compressive bearing capacity of the dynamic sensor.

Intex 2: The dynamic measuring range should be as wide as 0–500 kN. 

As mentioned in Index 1, it is the precondition for the sensor to have a high static compressive bearing capacity. While having a wide dynamic measuring range is the key function of the sensor. It is well known that the low-amplitude dynamic loads are not dangerous enough to induce landslides. Only when the measuring range is wide enough can the dynamic sensor monitor the dangerous high-amplitude disturbance. Therefore, based on the practical slope engineering projects [24,25,26,27,28], the measuring range of the sensor is set as 0–500 kN.

### 2.2. Key Techniques

#### 2.2.1. Theoretical Mechanism of Piezoelectric Sensors

**Figure 4:**
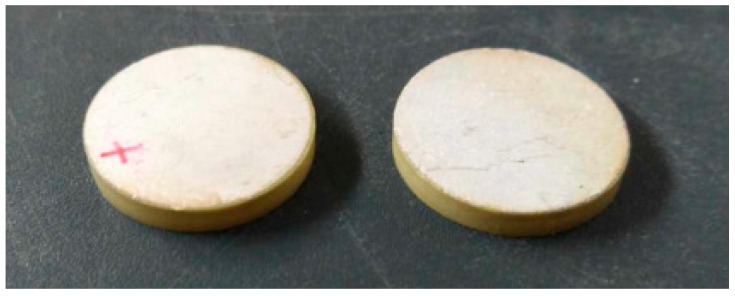
The positive and negative electrode surface of PZT-5 piezoelectric ceramic patches.

As the favorable characteristics of rapid dynamic response, high precision and good stability, the PZT-5 (Lead Zirconate-Titanate) piezoelectric ceramics, as shown in Figure 4, are selected as the basic force-sensing elements of the sensor. Thus, the sensor can also be called as piezoelectric sensor. Considering the high requirement of structural stability integral strength, the main body of the sensor will be steel-made. The high-strength steel-made main structure can also protect the brittle piezoelectric force-sensing elements in the sensor against the external concentrated load.

**Figure 5:**
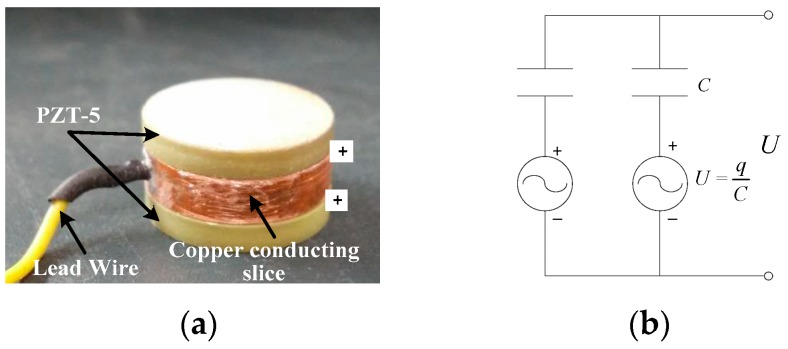
(**a**) The force-sensing element with two piezoelectric patches in parallel; (**b**) Its simplified equivalent circuit.

The working principle of piezoelectric ceramics is based on the piezoelectric effect [29]. That is, the piezoelectric ceramic will generate positive and negative charges in the upper and lower electrode surface when the external force is applied on it. The electric charges can be output as voltage signals through a conducting slice and a lead wire connecting with the positive electrode surface, as shown in Figure 5a. Due to the characteristic that the output voltage signals vary proportionally with the input signals of external forces, the piezoelectric ceramic is widely used as a force-sensing element to convert the physical quantity of force into voltage. The parameters of PZT-5 piezoelectric ceramics used in this paper are shown in Table 1 [29].

According to the piezoelectric effect, the single piezoelectric ceramic patch can be regarded as an equivalent series circuit with a voltage source and a capacitor. Figure 5 presents a type of common method of parallel connection with two piezoelectric patches and its corresponding equivalent circuit. Based on the principle of parallel circuit, the output voltage, *U*, can be obtained by calculating the *q*-to-*C* ratio of a single piezoelectric patch, where *q* denotes the positive electric charges and *C* the capacitance. The theoretical calculation formulas of *q* and *C* are given as

(1) $$\{\begin{array}{c} q=d_{33}F\\ C=\frac{\varepsilon_{r}\varepsilon_{0}S}{h} \end{array}$$

where *F* denotes the external force applied on the piezoelectric patch, *d*_33_ the piezoelectric constant of PZT-5 piezoelectric ceramics, *ɛ*_r_ and *ɛ*_0_ the relative and vacuum dielectric constant respectively, *S* the area of the electrode surface, and *h* the thickness of the piezoelectric patch.

Therefore, the functional relation between the output voltage, *U*, and the input external force, *F*, of the force-sensing element shown in Figure 5 is given as

(2) $$U=\frac{q}{C}=\frac{hd_{33}F}{\varepsilon_{r}\varepsilon_{0}S}$$

In addition, considering this piezoelectric sensor is to be embedded into the existing sensing system in the existing LMFS [24,25,26,27,28] as an improvement and supplement component, the design of the piezoelectric sensor should take the geometric dimensions of the adjacent existing devices into account. The parameters of the existing devices in the sensing system, namely, the vibrating wire sensor, the cable locket and the anchor cables are given in Table 2.

Combining the parameters in Table 1 and Table 2 and the theoretical calculating method based on Equations (1) and (2), we identify two key problems that need to overcome in the design of the piezoelectric sensor.

Firstly, the compressive strength of the PZT-5 piezoelectric ceramics is unable to directly satisfy the static ultimate compressive bearing capacity of 1500 kN arranged in Index 1.

**Figure 6:**
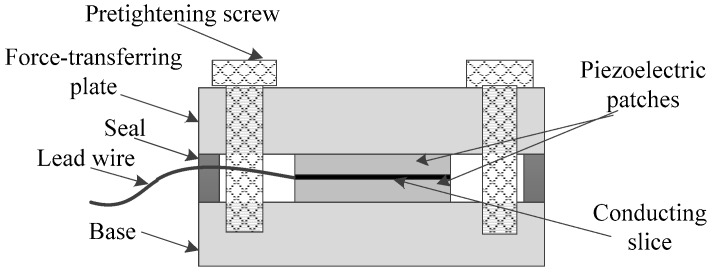
The simplified schematic of the common uniaxial piezoelectric sensor.

The simplified schematic of the common uniaxial piezoelectric sensor used in the engineering is shown in Figure 6. As can be seen, since the external load is mostly applied on the piezoelectric element through the force-transferring plate, the compressive capacity of the sensor directly depends on that of the piezoelectric patches. 

Considering the installation condition and the portability, the dimension of the piezoelectric sensor designed in this paper should be not more than that of the existing vibrating wire sensor (see Figure 1). Assume that the cross-sectional area of the piezoelectric sensor is 0.019 m^2^, which is slightly less than the existing sensor and close to the prototype shown in Section 3. Then we can multiply this value by the compressive strength of the PZT-5 piezoelectric ceramics given in Table 1. The product can hardly satisfy the loading requirement of Index 1. That is, as the limited compressive strength of the piezoelectric patch, the ultimate bearing capacity of the common piezoelectric sensor shown in Figure 6 can hardly match the high loading condition in slope engineering, even if the whole cross section of the dynamic sensor is fully covered with piezoelectric patches (Actually, the piezoelectric covering area cannot be greater than or equal to the cross-sectional area of the sensor).

Secondly, the direct output response voltage of the maximum dynamic load amplitude of 500 kN is too high to be collected and processed by the existing data acquisition equipment (DAE). According to the functional relation between the output voltage and the input external force, the value of *U* can be calculated approximately as 1500 V when substituting *F* as 500 kN and the other corresponding parameters in Table 1 into Equation (2). Obviously, this high-voltage response signal has greatly exceeded the measuring range of conventional DAEs and can even cause danger. 

Consequently, to ensure the piezoelectric sensor can satisfy Indexes 1 and 2 and overcome the two aforementioned problems, the two key techniques below are utilized. Technique 1 can implement the overload measuring ability of the sensor. That is, by using technique 1, the compressive strength of the sensor can be higher than the piezoelectric element. Technique 2 can quantitatively decrease the direct high response voltage by using electrical principle.

#### 2.2.2. Technique 1

The first technique is the self-structural pressure distribution method (SSPDM). The theoretical basis of SSPDM is the principle of stiffness distribution. That is, the total force can be distributed in accordance with the stiffness of the corresponding part. Based on the high compressive strength of steel, once the external force is mostly applied on the steel-made main body, the compressive strength of the piezoelectric sensor will improve considerably. By means of quantitative calculations, the total force can be rationally distributed and the component force on the main body, as well as that on the force-sensing elements can match the loading requirement of 1500 kN described in Index 1.

The theoretical formula of compressive/tensile stiffness is given as

(3) $$K=\frac{EA}{l}$$

where *E* denotes the elasticity modulus, *A* the effective area and *l* the length of the material.

**Figure 7:**
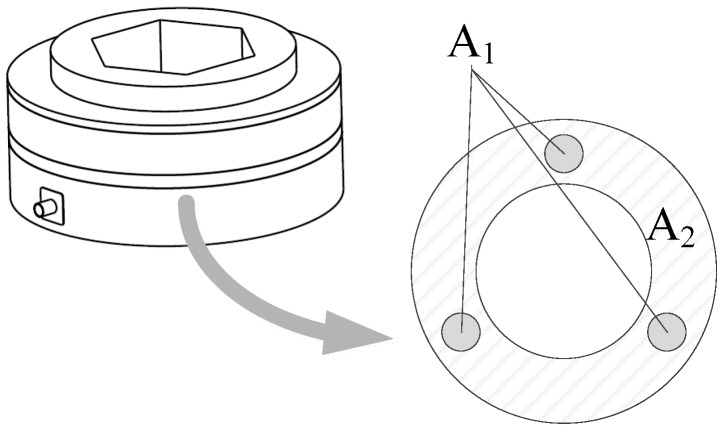
The force-bearing cross section of the sensor.

As can be seen in Figure 7, if the joints are not considered, the piezoelectric sensor can be regarded as a hollow cylinder and the force-bearing cross section is a hollow ring, which is similar to the vibrating wire sensor shown in Figure 1. *A*_1_ and *A*_2_ in the cross section denote the area of the force-sensing elements and the area of the steel main body, respectively. As *E* and *l* in Equation (3) are known, the key of the SSPDM is to acquire a rational *K*_1_-to-*K*_2_ ratio by adjusting the *A*_1_-to-*A*_2_ ratio and the *K*_1_-to-*K*_2_ ratio has to guarantee that the component force applied on low-strength piezoelectric force-sensing elements is lower than their total ultimate compressive bearing capacity. Subsequently, the *F*_1_-to-*F*_2_ ratio can be obtained based on the *K*_1_-to-*K*_2_ ratio. The above mentioned *K*_1_ and *K*_2_ refer to the stiffness of the piezoelectric force-sensing elements and the steel main body in the corresponding part of the sensor; *F*_1_ and *F*_2_ denote the component forces applied on the two structures, respectively.

**Figure 8:**
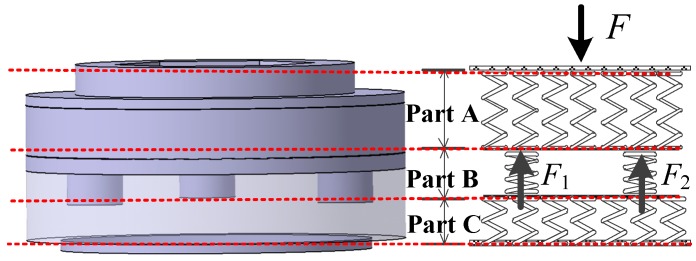
The equivalent spring model of the sensor.

Therefore, based on the mechanical characteristics, the piezoelectric sensor can be regarded as a series of springs connected in parallel and serial patterns shown in Figure 8. As the springs in Parts A, B, and C are connected in series, the force acting on each part is equal to the external force *F*. Parts A and C stand for the steel-made and non-sensing parts of the sensor, which can satisfy the design requirements obviously. The force-sensing elements are placed in Part B which is composed of two springs, whose stiffness is respectively in accordance with *K*_1_ and *K*_2_ and corresponding to *A*_1_ and *A*_2_ in Figure 7. Thus, *F*_1_ and *F*_2_, the corresponding component forces respectively on the force-sensing elements and the main body, can be calculated as follows.

As the two springs in Part B are connected in parallel, the sum of the two component forces, *F*_1_ and *F*_2_, is equal to the total external force *F*. In addition, *F*_1_ and *F*_2_ are directly proportional to *K*_1_ and *K*_2_, respectively, which can be expressed as

(4) $$\{\begin{array}{c} F_{1}=\frac{K_{1}F}{K_{1}+K_{2}}\\ F_{2}=\frac{K_{2}F}{K_{1}+K_{2}} \end{array}$$

Considering the proportional relation between *F*, the total external force, and *F*_1_, the component force which should less than the compressive capacity applied on the force-sensing elements, the advisable *K*_1_-to-*K*_2_ ratio can be calculated and ensure the piezoelectric sensor can satisfy Index 1.

#### 2.2.3. Technique 2

The second technique is the capacitive circuit voltage distribution method (CCVDM). The theoretical basis of CCVDM is the principle that in the circuit with two capacitors connected in series, the voltage across the capacitor has an inversely proportional relationship to its capacitance. Thus, by collecting the voltage across one specific capacitor in the circuit as the final output signal, the output voltage of the sensor can be quantitatively decreased. 

Actually, based on Equations (3) and (4), the final output voltage can be directly controlled to meet the requirement of the DAE’s measuring range. However, this one-step method is unadvisable because the sensitivity of the sensor will be limited to be ultra-low, and consequently the signal-to-noise ratio will be sharply reduced.

**Figure 9:**
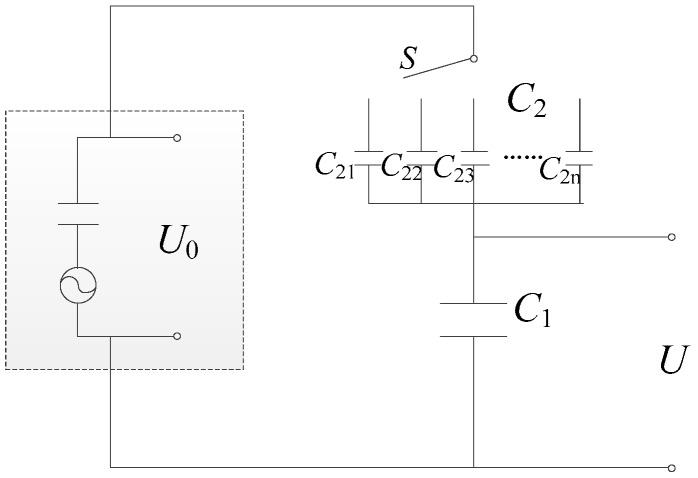
The voltage-reduced capacitive circuit.

In this situation, a multistage switching capacitive circuit, as is shown in Figure 9, is designed. Using this circuit, the direct output voltage of the force-sensing elements can be distributed into two components with the certain proportional ratio. Thus, it can guarantee that one of the components can be collected by the DAE as the final voltage signals of the piezoelectric sensor.

Specifically speaking, as can be seen in Figure 9, *U*_0_ denotes the voltage directly output by the force-sensing elements. *U* is the voltage across *C*_1_, as well as the final output voltage of the sensor. *C*_1_ is a capacitor of relatively high capacitance and *C*_2_ are a set of capacitors of low capacitance connected in parallel. Each element of *C*_2_, named from *C*_21_ to *C*_2n_, has a given proportional relation to *C*_1_, such as 1/2, 1/5, 1/10, 1/100, and the other ratios.

The relation between the final output voltage, *U*, and the direct responding voltage, *U*_0_, in Figure 9, can be given as

(5) $$U=\frac{C_{2}U_{0}}{C_{1}+C_{2}}$$

As the voltage measuring range of the DAE is generally known and the maximal amplitude of the measuring disturbing force has been prearranged in Index 2 of Section 2.1, considering the proportional relation between *U*_0_, which can be theoretically obtained by Equations (2)–(4), and the voltage measuring range of DAE, the advisable *C*_2_-to-*C*_1_ ratio can be selected by the regulating switch *S*. Thus, the final output voltage of the piezoelectric sensor for the ultimate amplitude of the dynamic load can be collected.

## 3. Assembly of the Sensor

**Figure 10:**
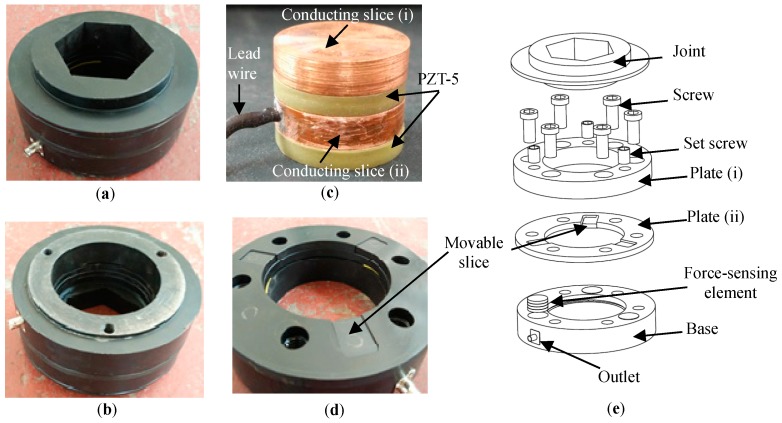
The prototype and the main components of the sensor. (**a**) The front side and (**b**) back side of the piezoelectric sensor; (**c**) The force-sensing element in the sensor; (**d**) The installation state of the movable slices; (**e**) The exploded-views of the sensor.

**Figure 11:**
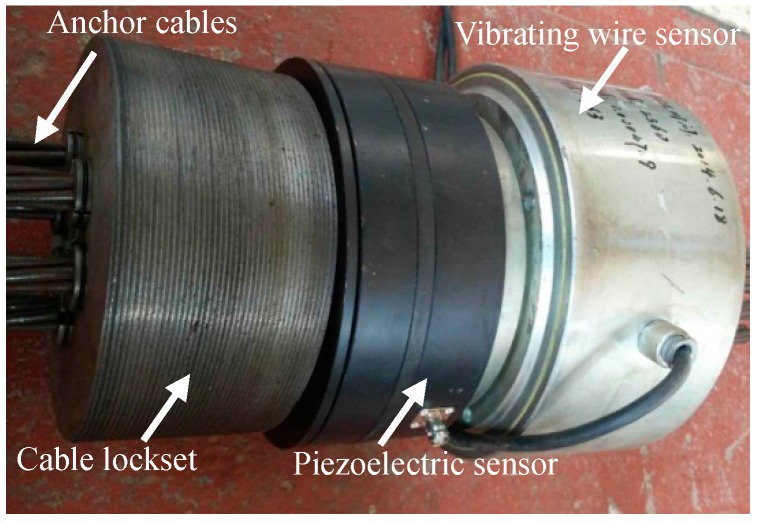
The connection form of the sensing system.

Figure 10 shows the prototype and the main components of the piezoelectric sensor. The connection pattern of the sensors in the sensing system is depicted in Figure 11. As can be seen in Figure 10a,b, the protruding blocks on the front and back of the piezoelectric sensor are utilized to embed into the vibrating wire sensor and the cable lockset respectively, as is shown in Figure 11. Three force-sensing elements are inside the piezoelectric sensor. Each element shown in Figure 10c comprises two PZT-5 piezoelectric patches and two copper conducting slices with the same cross section. As Figure 10c shows, the positive electrodes of the two piezoelectric patches are both connected by the copper conducting slice (ii), on which a lead wire is attached. The connection method shown in Figure 10c is widely used in the practical application due to the advantages such as improving the signal-to-noise ratio and eliminating the extra insulation between the positive electrode and the basic structure. All the output voltage signals of the three force-sensing elements are converged at the outlet of the piezoelectric sensor via the lead wires placed into the ring-like groove in the base. Before being collected, the final output voltage signals are regulated by the embedded capacitive circuit shown in Figure 9.

Six screws and three set screws are used for the assembly of the piezoelectric sensor, which are shown in Figure 10e. As can be seen, plate (i), plate (ii), and the base are fastened by the six screws with uniform angles. The three set screws are bolted into plate (i). The three moving slices, each of which is thinner than plate (ii), are butted on the top of the force-sensing elements by the heads of the set screws, respectively. The height of the single force-sensing element is designed to be slightly higher than the depth of corresponding cylindrical groove in the base and the height of the movable slice is lower than that of the plate (ii) when plate (ii) is placed on the top surface of the base, as can be seen in Figure 10d. 

Thus, most of the external pressure applied on plate (i) will be transferred onto plate (ii), while only a small part onto the movable slices as well as the force-sensing elements. Due to the high compressive strength of the basic steel-made structure, including the plate (i), plate (ii), the base and the other components, the sensor can bear the high pressure exceeding the compressive strength of piezoelectric ceramic and reach what the Index 1 requires.

It is worth mentioning that all the screws including the six screws and the three set screws should be fully tightened and all the connection gaps among the components of the sensor, as well as the exposed grooves in the relative components should be sealed up to isolate from the outer moist. Besides, as the PZT-5 piezoelectric ceramic is brittle and easily damaged by concentrated force, the contact of the PZT and the adjacent components, such as the conducting slice (i) and (ii), should be plane-to-plane type.

With the connection between the outlet of the piezoelectric sensor and the DAE via a data wire, the response voltage signals to the changing external forces can be obtained. The DAE used in this paper is the LMS Spectrum Testing System developed by Belgium LMS Co.

The parameters of the piezoelectric sensor and its main components are presented in Table 3, based on which, it can be theoretically verified whether the sensor can meet the Indexes 1 and 2 by using the two techniques of SSPDM and CCVDM in Section 2.2.

**Figure 12:**
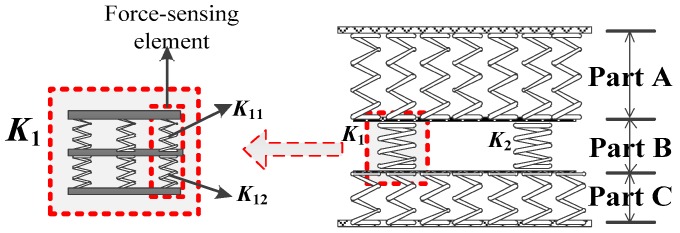
The equivalent spring model of the single force-sensing element.

Figure 12 presents the equivalent spring model of the piezoelectric sensor based on Figure 10, where *K*_1_ in Part B denotes the total stiffness of the three force-sensing elements in parallel. Each element contains two piezoelectric patches, whose total stiffness is *K*_11_, and two copper conducting slices, whose total stiffness is *K*_12_. Then *K*_1_, *K*_11_ and *K*_12_ can be obtained as

(6) $$K_{1}=3\frac{K_{11}K_{12}}{K_{11}+K_{12}}$$

(7) $$K_{11}=\frac{E_{11}A_{11}}{l_{11}};\;K_{12}=\frac{E_{12}A_{12}}{l_{12}}.$$

where *E*_11_ and *A*_11_ are the elastic modulus and the electrode surface area of the PZT-5 piezoelectric patch, respectively, which can be acquired in Table 1 as 117 MPa and 314 mm^2^. Similarly, *E*_12_ and *A*_12_ are those of the copper conducting slices, which are 100 MPa and 314 mm^2^. *l*_11_ and *l*_12_ mean the total height of the two piezoelectric patches and the two copper electrode slices, respectively, which are 6 mm and 10 mm. Thus, *K*_1_ can be obtained as 6.12 × 10^9^ N/m.

Meanwhile, *K*_2_, the total stiffness of the steel main body in Part B can also be calculated as 2.1 × 10^11^ N/m when substituting the corresponding parameters in Table 3 into Equation (3).

As a result, when the piezoelectric sensor is bearing external ultimate static load of 1500 kN and transient force amplitude of 500 kN, the component forces applied on the force-sensing elements, *F*_1_, can be respectively calculated as 43.23 kN and 14.41 kN by using Equation (4). The former is less than the total ultimate compressive capacity of the three piezoelectric force-sensing elements, which can be obtained by the product between the parameters of compressive strength and cross sectional area shown in Table 1. The latter can be used to calculate the response voltage via Equation (2) and the theoretical solution is 1025 V, which is more than 100 times greater than the regular signals since the maximal measuring range of the LMS Spectrum Testing System is within 10 V. Therefore, the advisable *C*_2_-to-*C*_1_ ratio in the capacitive circuit shown in Figure 9 can be selected as 1/200, so that the final output voltage for the ultimate transient load is 5.01 V and the theoretical sensitivity coefficient of the sensor is 0.01 V/kN.

## 4. Calibration Experiments

Conducting the calibration experiments and acquiring the sensitivity are absolutely necessary for the sensors before their practical engineering applications. The purpose of the calibration for the sensors [11,12,13,14,15,16,17,18,19,20,21,22,23,24,25,26,27,28], as well as the sensor in this paper, is similar, which is to obtain the relation between the input physical parameter, such as force, and the output parameter, such as voltage. However, the requirements of the calibration experiments are different, which depend on the practical application environments.

For the sensor in this paper, the calibration experiments should as closely as possible simulate the actual static and dynamic loading conditions in slope engineering. Thus, the static and dynamic characteristics of the sensor can also be verified. However, considering the severe stress conditions in practical slope engineering, conventional testing machines can hardly match the high requirements. For instance, the drop hammer impact tester can provide only dynamic load (without static preload); the fatigue tester can provide both static preload and dynamic load, but the loading amplitudes are limited. Therefore, this section presents an independently invented static and dynamic loading mechanism, which can provide step-load with different amplitudes on the basis of the preload. Thus, it can meet the high static and dynamic loading requirements.

For the perspective of frequency-domain analysis, the step-load is a typical wide-frequency range exciting signal. The steeply rising stage and its peak value of the step signal are mainly composed of the high-frequency components, while the platform stage is low-frequency components [30,31]. Then, using the step-load with different amplitudes as the input excitation to calibrate the sensor can mostly eliminate the nonlinear response errors caused by the low-frequency piezoelectric charge leakage. Thus, it is advisable to employ the wide-frequency step-load for the calibration of the sensor. However, the common defect of low-frequency measuring errors for the piezoelectric sensor cannot be avoided in the practical engineering application, since the dynamic disturbing signals are various, containing the low-frequency disturbance certainly. The low-frequency correction method will be specially introduced in Section 5. 

### 4.1. Experimental Setup

**Figure 13:**
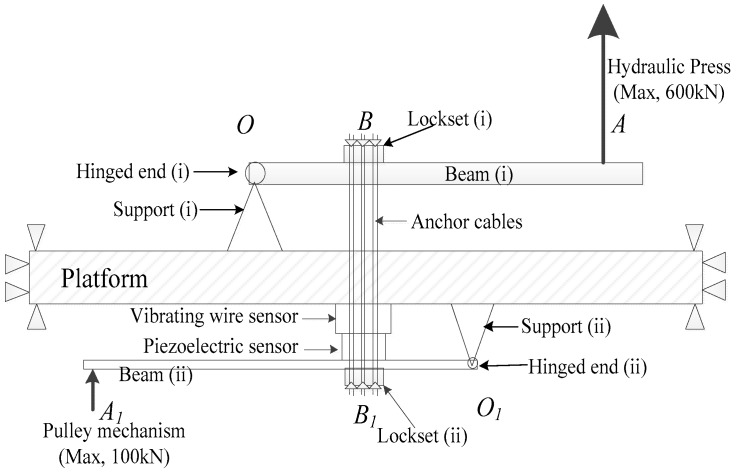
Schematic of the static and transient loading mechanism.

**Figure 14:**
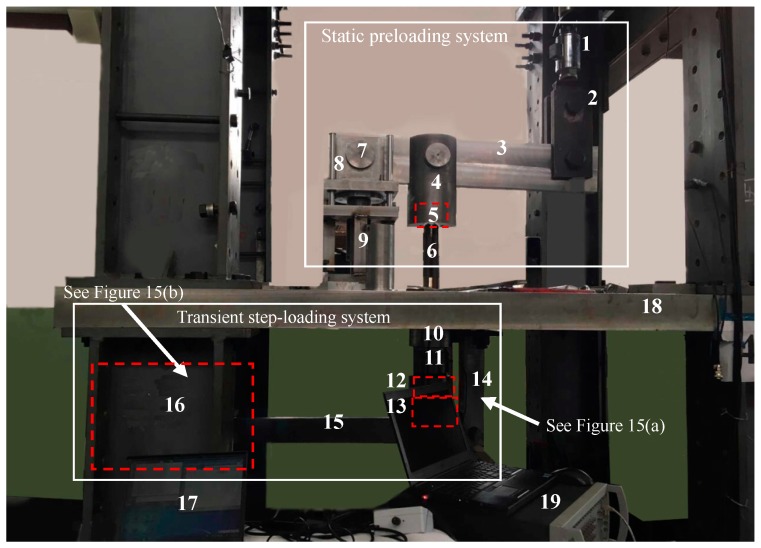
Experimental setup. (**1**)-Hydraulic Press; (**2**)-Lug (iii); (**3**)-Beam (i); (**4**)-Lug (ii); (**5**)-Lockset (i); (**6**)-Anchor cables; (**7**)-Hinged end; (**8**)-Lug (i); (**9**)-Support (i); (**10**)-Vibrating wire sensor; (**11**)-Piezoelectric sensor; (**12**)-Lockset (ii); (**13**)-Lug (iv); (**14**)-Support (ii); (**15**)-Beam (ii); (**16**)-Pulley mechanism; (**17**)-DAE of vibrating wire sensor; (**18**)-Platform; (**19**)-LMS system.

As can be seen in Figure 13 and Figure 14, the whole loading mechanism comprises a preloading system and a step-loading system, which can respectively provide the high enough static preload and the transient load with certain amplitudes. The preloading system is located upon the platform, which contains the components of the Hydraulic Press, beam (i), support (i), the lugs and the pins, while the step-loading system under the platform is composed of the quick-release hook, beam (ii), support (ii), the matching lugs and the pins, and the other components. By the pinned connections, both the upper and lower loading systems can be regarded as levy mechanisms.

Both the two loading systems provide load on the sensing system through the anchor cables, where the sensing system and its connecting pattern are shown in Figure 11. The anchor cables are made up of six steel strands. As a kind of hollow structure, each of the sensing components including the piezoelectric sensor, the vibrating wire sensor and the cable locksets is crossed in the middle by the anchor cables. Cable lockset (i) is clamped at the top of the anchor cables and cable lockset (ii) is at the bottom. The two locksets are also fixedly connected with and inside lug (ii) and lug (iv), respectively. Thus, the whole sensing system can be compacted by lockset (ii) under the platform when applying load as follows.

At the first step for performing the static preload, *A*, the free end at the right of the beam (i), can be slowly pulled up by the Hydraulic Press, which can provide both uniaxial tension and compression force, as is shown in Figure 14. Then, *B* is consequently raised since *O* at the left hinged end of beam (i) can be considered as the hinged support of the levy mechanism. As a result, the lockset (i) stretches upward the anchor cables. Thus, the sensing system including the piezoelectric sensor and the vibrating wire sensor is tightly compressed by lockset (ii). By monitoring the reading of the vibrating wire sensor, the static preload can be controlled to reach the design values.

**Figure 15:**
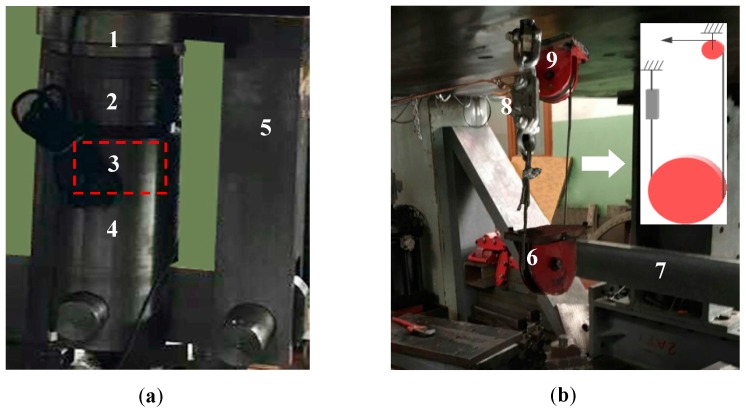
(**a**) The sensors and the adjacent devices shown in Figure 14; (**b**) Pulley mechanism shown in Figure 14. (**1**)-Vibrating wire sensor; (**2**)-Piezoelectric sensor; (**3**)-Lockset (ii); (**4**)-Lug (iv); (**5**)-Support (ii); (**6**)-Pulley (i); (**7**)-Beam (ii); (**8**)-Quick-release hook; (**9**)-pulley (ii).

The second step is to provide transient step-load on the basis of the first step, which can be divided into two substeps. Firstly, *A*_1_, the free end at the left of beam (ii), can be quasi-statically pulled up by the pulley mechanism shown in Figure 15b. Thus, the total compression force applied on the sensing system is further increased. That is, an additional part of preload is applied on the sensing system by the lower loading system in this substep. By monitoring the reading of the vibrating wire sensor, this additional preload can be quantitatively controlled. Secondly, using the quick-release hook, the additional preload applied on the sensing system in the first substep can be unloaded transiently. Thus, the step-load with the specific amplitude is achieved via the lower loading system.

Meanwhile, the response voltage of the piezoelectric sensor can be recorded by the LMS Spectrum Testing System during the action of the step-load. With the output response voltage Δ*U*, and the input specific amplitude of step-load Δ*F*, the sensitivity coefficient of the piezoelectric sensor *α* can be obtained, that is,

(8) $$\alpha =\frac{\Delta U}{\Delta F}$$

In particular, the maximum tension that the Hydraulic Press can provide is 600 kN and the ultimate bearing capacity of the quick-released hook is 5 t (almost 50 kN) so that the pulley mechanism shown in Figure 15b can provide tension of 100kN. As is shown in Figure 13, the length of *OA* is designed as 2 times more than *OB* and *O*_1_*A*_1_ is 5 times more than *O*_1_*B*_1_. Based on the leverage principle, the upper and lower loading system shown in Figure 13 and Figure 14 can apply enough high static load of 1800 kN and transient load with amplitude of 600 kN , which can satisfy the requirements expressed in Indexes 1 and 2, respectively.

It is also worth mentioning that the strength and stiffness of all the components in the loading mechanism have been theoretically verified and qualified under the ultimate static and dynamic conditions.

### 4.2. Experimental Scheme

Table 4 presents the scheme of the calibration experiments, which can be divided into four sets according to the different static preload levels, namely, 300 kN, 600 kN, 1000 kN and 1500 kN. Each set can be further classified into several subsets based on the amplitude of the step-load. Take subset 1-1 as an example. The first step is to apply the static preload of 300 kN on the sensing system, while at the second, the transient step-load with the amplitude of 50 kN will be applied.

### 4.3. Experimental Results

**Figure 16:**
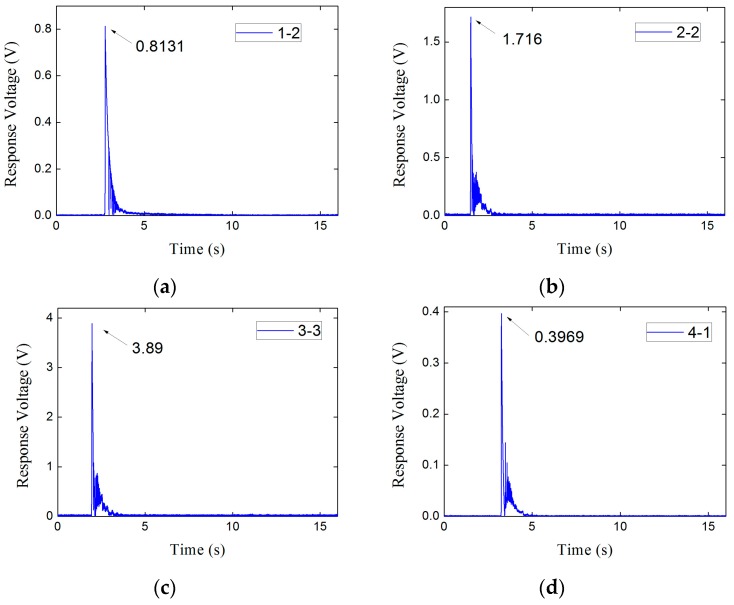
The response voltages. of subsets. (**a**) Subset 1-2; (**b**) Subset 2-2; (**c**) Subset 3-3; (**d**) Subset 4-1.

The peak response voltage of every subset is also reported in Table 4. Considering the limited space and the similarity of the response signals to the step-load, only the subsets 1-2, 2-2, 3-3 and 4-1 in every set are given, as is shown in Figure 16.

**Figure 17:**
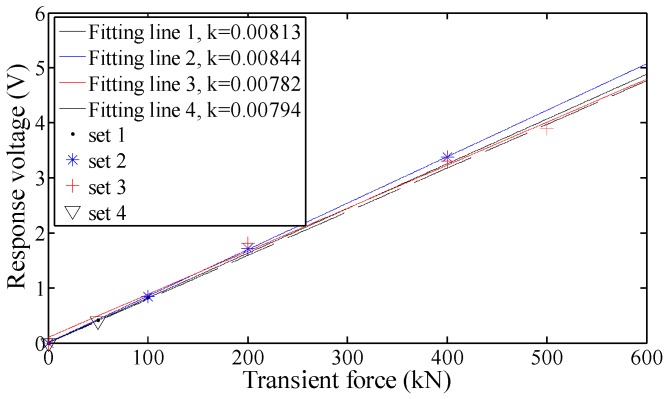
Linear fitting results of the four sets shown in Table 4.

By means of the linear fit, the fitting lines of the experimental results can be obtained in Figure 17. As can be seen, the piezoelectric sensor has a satisfactory wide range of linearity, since the goodness of fit of the each fitting straight line is more than 96%. The sensitivity coefficient of the sensor can be acquired as 0.0081 V/kN by calculating the average slope of the fitting straight lines.

In addition, all of the fitting straight lines are almost coincident with each other and their slopes are quite similar. It indicates the piezoelectric sensor has a stable sensitivity under different preload levels.

Particularly, the subset 4-1 shown in Table 4 can be used to check the static ultimate compressive bearing capacity of the piezoelectric sensor, since the peak static load has reached the static index of 1500 kN. As is shown in Figure 16d, the response result of subset 4-1 have the consistent property with the other sets and follows the linear law shown in Figure 17. It illustrates that the sensor maintains the good behavior under the ultimate preload condition. Therefore, the piezoelectric sensor has satisfied the static-load requirement of Index 1.

Besides, the amplitude of the transient load in subset 3-3 is 500 kN, which has reached maximum of the dynamic measuring index, the response result in Figure 17 shows that it also follows the linear law with the other subsets. That is, the measuring range of the piezoelectric sensor is as wide as 500 kN at least, which is enough to satisfy Index 2.

Therefore, both the prearranged Indexes 1 and 2 for the piezoelectric sensor has been well satisfied experimentally.

It is noteworthy that the experimental sensitivity coefficient of the piezoelectric sensor is less than 20% with the theoretical result, which can be acceptable when considering the error source of the limited piezoelectric property and machining precision. Moreover, the coherence of the theoretical and experimental results can also verify the helpfulness and effectiveness of techniques 1 and 2 in Section 2.2.

## 5. Correction of Low-Frequency Measuring Signals

Piezoelectric sensors have the natural defect that the phenomenon of charge leakage can cause remarkable measuring error for the low-frequency measuring signals. Thus, in the slope engineering, the measuring result directly calculated based on sensitivity coefficient of Equation (8) for the low-frequency disturbance will be inaccurate.

The correction method is conducted in frequency domain, since the error mainly concentrates in low frequency range [30,31]. The low-frequency correction principle, the experimental verification and the correction results are introduced as follows.

### 5.1. Low-Frequency Correction Principle

The differential equation on the output voltage, *U*(*t*) , and the external acting force, *F*(*t*), of the piezoelectric sensor taking time *t* as the variable can be expressed as [30,31]

(9) $$RC\frac{d\left[U\left(t\right)\right]}{dt}+U\left(t\right)=d_{33}RA\frac{d\left[F\left(t\right)\right]}{dt}$$

where *d*_33_ denotes the piezoelectric constant, *A* the total working area of the piezoelectric element of the sensor, *R* and *C* the equivalent resistance and capacitance respectively.

The expression in frequency domain of Equation (9) can be obtained by Fourier transform as

(10) $$\left(i\omega RC+1\right)U\left(\omega \right)=i\omega d_{33}RAF\left(\omega \right)$$

where i is the imaginary unit, *ω* the angular frequency. 

Thus, based on Equation (10), the function relationship in frequency domain of the actual external acting force, *F*_a_(*ω*), and the output response voltage, *U*(*ω*), can be written as

(11) $$F_{a}\left(\omega \right)=\frac{U\left(\omega \right)}{\alpha }\cdot \frac{1+i\omega \tau }{i\omega \tau }$$

where *α* = *d*_33_*A*/*C* and *τ* = *RC* are the theoretical expressions of the sensitivity coefficient and discharge time constant of the piezoelectric sensor, respectively. Both *α* and *τ* are constants, theoretically.

Generally, the sensitivity coefficient, *α*, is defined based on the Equation (8) in the calibration experiments. Then the measuring value of the external acting force, *F*_m_(*ω*), can be acquired as

(12) $$F_{m}\left(\omega \right)=\frac{U\left(\omega \right)}{\alpha }$$

Obviously, errors exist between *F*_m_(*ω*) and *F*_a_(*ω*), when comparing Equations (11) and (12). Thus, the *F*_m_(*ω*) needs to be corrected by using the correction function, *C*(*ω*), to obtain the correctional result, *F*_c_(*ω*), which would be consistent with the actual external acting force, *F*_a_(*ω*), that is

(13) $$F_{a}(\omega )=F_{c}(\omega )=F_{m}(\omega )C(\omega )$$

Substituting Equations (11) and (12) into Equation (13), the correction function *C*(*ω*) can be rewritten as

(14) $$C(\omega )=\frac{1+i\omega \tau }{i\omega \tau }$$

As can be seen from Equation (14), when the value of *ω* is relatively large, *C*(*ω*) is close to1 and play a very weak correctional role for *F*_m_(*ω*) in Equation (12); when *ω* is small, *C*(*ω*) will exert significant influences for *F*_m_(*ω*). It is preliminary verified the effectiveness of this low-frequency correction method.

The constant parameters of *α* and *τ* in Equations (11)–(14) can be identified as follows.

### 5.2. Parameter Identification of the Correction Function

As can be seen in Figure 16, the voltage response signals in time domain of step force applied on the piezoelectric sensor are presented as an exponential decay model, that is

(15) $$U\left(t\right)=F_{i}\alpha e^{-\frac{t}{\tau }}$$

where *F_i_* denotes the amplitude of the step force, *α* and *τ* the sensitivity coefficient and discharge time constant of the piezoelectric sensor.

**Figure 18:**
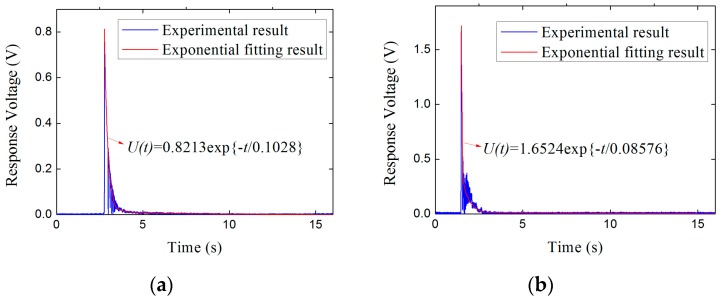

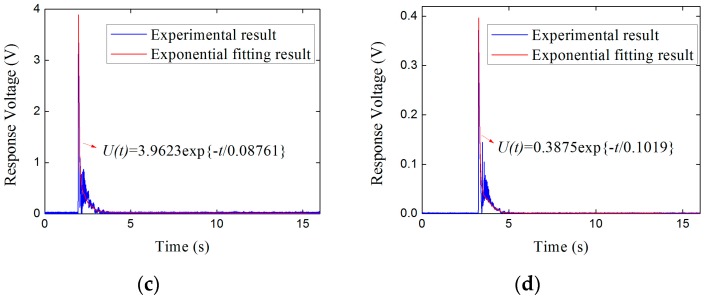
Exponential fitting results of the response voltages. (**a**) Subset 1-2; (**b**) Subset 2-2; (**c**) Subset 3-3; (**d**) Subset 4-1.

Each voltage response result in time domain of the subsets shown in Table 4 in Section 4.2 can be exponential fitted. In view of the limited space, Figure 18 only shows the fitting results corresponding to the subsets in Figure 16.

Then, based on the exponential fitting results, the values of *α* and *τ* in Equation (15) are obtained by calculating the mean of all subsets, that is

(16) $$\alpha =\frac{1}{n}\sum_{i=1}^{n}\alpha_{i}=0.00804\;V/\mathrm{kN};\;\tau =\frac{1}{n}\sum_{i=1}^{n}\tau_{i}=0.09452\;s$$

We can find that the values of sensitivity coefficient *α* obtained in Equations (16) and (8) in the section of calibration experiments are quite the same. Substituting Equation (16) into Equation (14), the final expression of the correction function is obtained

(17) $$C(\omega )=\frac{1+0.09452i\omega }{0.09452i\omega }$$

### 5.3. Experimental Verification of the Low-Frequency Correction Method

To verify the low-frequency correction method, the measuring results of the step force and harmonic force with the known amplitudes applied on the piezoelectric sensor are corrected by using the correction function shown in Equation (17).

**Figure 19:**
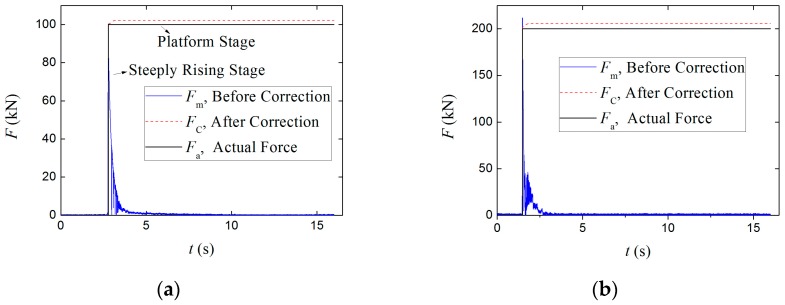
(**a**) Comparisons of *F*_m_, *F*_c_ and *F*_a_ corresponding to subset 1-2; (**b**) Comparisons of *F*_m_, *F*_c_ and *F*_a_ corresponding to subset 2-2.

**Figure 20:**
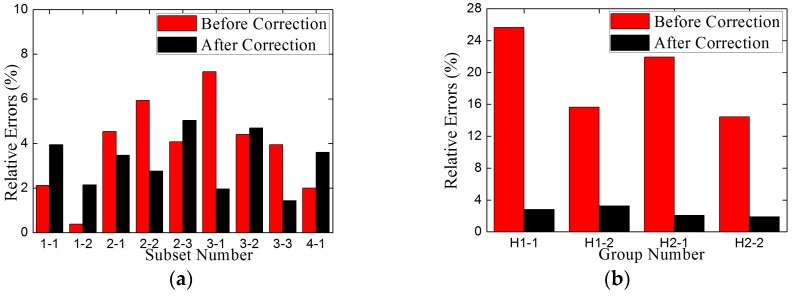
(**a**) The relative measuring errors for step force before and after correction; (**b**) The relative measuring errors for harmonic force before and after correction.

For the low-frequency correction of the measuring results of the piezoelectric sensor under the step force with different amplitudes, the amplitudes of the direct measuring results before correction, *F*_m_, the correctional results after correction, *F*_c_, and the actual external force, *F*_a_, are shown in Table 5. Due to the limited space, only the results corresponding to subsets 1-2 and 2-2 are shown in Figure 19. The relative errors between *F*_m_ and *F*_a_, *F*_c_ and *F*_a_ are also tabulated in Table 5, which are also summarized in Figure 20a.

As can be seen in Table 5 and Figure 20a, the amplitudes of the measuring results for step force after correction, *F*_c_, are basically consistent with those before correction, *F*_m_, as well as the amplitudes of actual external force, *F*_a_, since the relative errors between *F*_m_ and *F*_a_, *F*_c_ and *F*_a_ are similar. The errors of *F*_m_ are from 0.38% to 7.22%, while the *F*_c_ from 1.44% to 5.03%.

As can be seen in Figure 19, the differences between *F*_m_ and *F*_c_ are concentrated in the platform stage, that is, the low-frequency correction effect is mainly reflected in the platform stage. As a result, the curves of *F*_c_ and *F*_a_ in this stage are quite close. It indirectly proves that the steeply rising stage and its peak value of the step signal are mainly composed of the high-frequency components, while the platform stage is low-frequency components. It can also prove that the calibration method for piezoelectric sensor to acquire the sensitivity coefficient through obtaining the ratio of the peak response voltage to the amplitude of wide-frequency step force shown in Equation (8) in Section 4 is correct and advisable.

**Figure 21:**
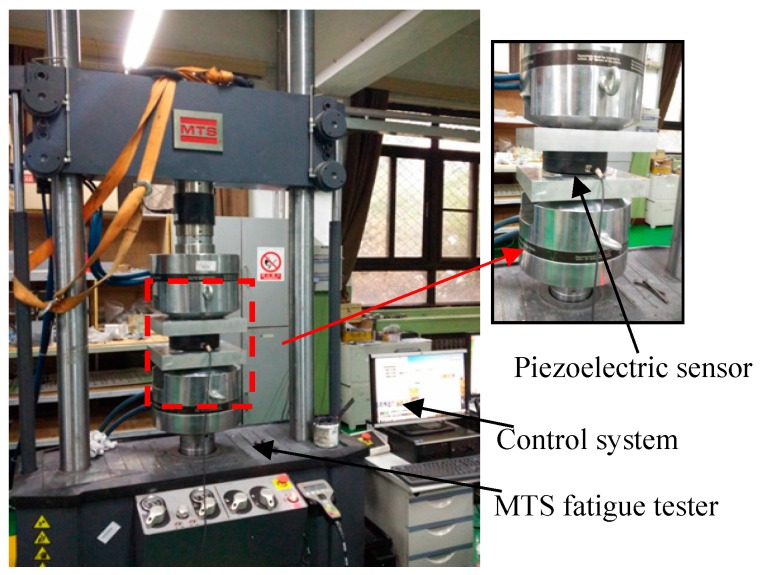
MTS fatigue tester for low-frequency harmonic force experiments.

**Figure 22:**
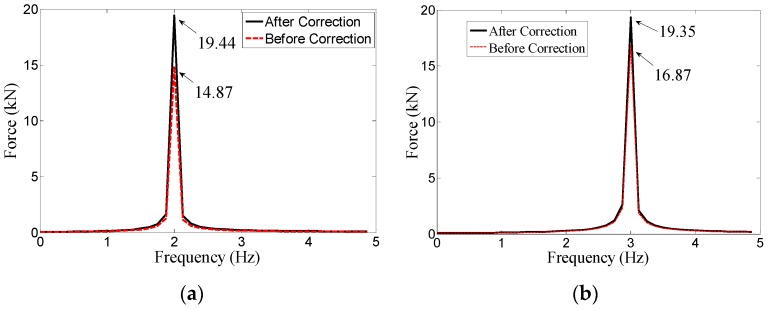
(**a**) The frequency-domain results of H1-1 before and after correction; (**b**) The frequency-domain results of H1-2 before and after correction.

**Figure 23:**
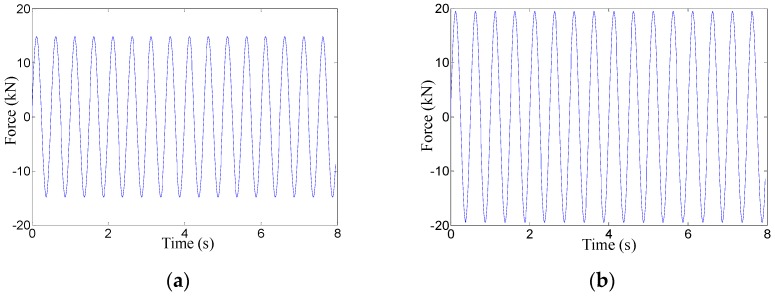
(**a**) The time-domain results of H1-1 before correction; (**b**) The time-domain results of H1-1 after correction

For the low-frequency correction of the measuring results of the piezoelectric sensor under the harmonic force, the MTS fatigue testing machine shown in Figure 21 is used to provide harmonic force, whose loading scheme is reported in Table 6. The comparisons of *F*_m_, *F*_c_ and *F*_a_ and the relative errors between *F*_m_ and *F*_a_, *F*_c_ and *F*_a_ are also tabulated in Table 6, as well as illustrated in Figure 20b. Considering the limited space, only the frequency-domain results before and after correction of H1-1 and H1-2 are shown in Figure 22 and the time-domain results before and after correction of H1-1 are shown in Figure 23. 

As can be seen in Table 6 and Figure 20b, the relative measuring errors before correction of the piezoelectric sensor for low-frequency harmonic signals are sharply decreased after correction. The measuring results after correction are quite the same with the actual external force.

Therefore, based on the results in Table 5 and Table 6 as well as in Figure 19, Figure 20, Figure 22 and Figure 23, this low-frequency correction method for the piezoelectric sensor is validated to be effective for the measurement of low-frequency signals. It can broaden the measuring frequency-band and strengthen the measuring reliability of the piezoelectric sensor for dynamic monitoring in slope engineering.

It is worth noting that the algorithm of the low-frequency correction method should be embedded into the DAE as a signal processing module to improve the measuring accuracy of the sensor. However, as it is not the key research scope in this paper, the physical realization of the algorithm module as well as the whole DAE is not mentioned.

## 6. Conclusions

This paper presents a piezoelectric sensor for the dynamic force monitoring of landslide, which can complement the measuring ability of the existing LMFS in slope engineering. The following key conclusions are obtained:

(1) Two techniques of SSPDM and CCVDM are employed in the design of the sensor to satisfy the two prearranged static and dynamic indexes proposed based on the severe stress conditions in slope engineering. The SSPDM can greatly improve the compressive capacity (up to 1500 kN). The CCVDM can quantitatively decrease the high direct response voltage, by which the response signal of the sensor for the amplitude dynamic load of 500 kN can be collected.
(2) The calibration experiments are conducted using the static and transient loading mechanism, which is independently developed employing the lever principle and can match the high loading requirements in practical engineering. Based on the experimental results, the sensitivity coefficient is obtained. The experimental results also reveal that the sensor has compressive capacity up to 1500 kN, stable sensitivities under different static preload levels and wide-range dynamic measuring linearity from 0 to 500 kN.
(3) The low-frequency correction method for the piezoelectric sensor is proposed and experimentally verified by imposing the step force and low-frequency harmonic force with different amplitudes on the sensor. The results reveal that the relative errors after correction are much lower than those before correction. Thus, low-frequency measuring reliability of the piezoelectric sensor is effectively improved.

Therefore, due to the excellent behavior of high compressive bearing capacity, wide dynamic measuring range and the improving low-frequency measuring reliability, the piezoelectric sensor invented in this paper can be embedded into the existing LMFS as a complement for dynamic monitoring.

It is worth noting that the sensor presented in this paper is specialized to monitor the dynamic force of slope engineering, but the principle of the design of the sensor and the calibration mechanism with high loading requirements, as well as the low-frequency correction method for piezoelectric elements can be also used to other relevant fields. For instance, the SSPDM can also be extended to the design of the sensors with higher strength and wider measuring range in the large engineering projects, while the CCVDM can be also used to control voltage-signal amplitude in the electrical fields.

## Figures and Tables

**Table 1:** Parameters of PZT-5 piezoelectric ceramic.

Parameter Name	Value	Units
Electrode surface area	3.14 × 10^−4^	m^2^
Thickness	0.003	m
Density	7.5 × 10^3^	kg/m^3^
Elastic modulus	117	GPa
Compressive strength	76	MPa
Piezoelectric constant	4.15 × 10^−7^	C/N
Relative dielectric constant	2100	-
Vacuum dielectric constant	8.85 × 10^−12^	F/m

**Table 2:** Geometric parameters of the vibrating wire sensor, the cable locket, and the anchor cables.

Parameter Name		Value	Units
Vibration wire sensor	Outer diameter	0.066	m
	Inner diameter	0.111	m
	Height	0.1	m
Cable lockset	Diameter	0.172	m
	Height	0.06	m
Anchor cables	Outer diameter	0.092	m
	Diameter (single cable)	0.01524	m
	Cable quantity	6	-

**Table 3:** Parameters of the piezoelectric sensor and its main components.

Parameter Name		Value	Units
Piezoelectric sensor	Outer diameter	0.19	m
	Inner diameter	0.11	m
	Height	0.073	m
Base	Thickness	0.035	m
	Depth of cylindrical groove	0.0155	m
Plate (i)	Thickness	0.025	m
Plate (ii)	Thickness	0.008	m
	Basal area	0.016	m^2^
Moving slice	Thickness	0.006	m
Force-sensing element	Height	0.016	m
Copper conducting slice	Thickness	0.005	m
	Elastic module	100	Gpa
Steel (main body)	Elastic module	210	Gpa

**Table 4:** Experimental scheme and peak response voltage of every subset.

Set	Static Preload/kN	Subset	Amplitude of Transient Step-Load/kN	Peak Response Voltage/V
1	300	1-1	50	0.4163
		1-2	100	0.8131
2	600	2-1	100	0.8467
		2-2	200	1.716
		2-3	400	3.372
3	1000	3-1	200	1.737
		3-2	400	3.097
		3-3	500	3.890
4	1500	4-1	50	0.3969

**Table 5:** Measuring errors before and after correction of the piezoelectric sensor for step force.

Set	Subset	Amplitude of *F*_a_/kN	*F*_m_ (Before Correction)		*F*_c_ (After Correction)	
			Amplitude/kN	Error	Amplitude/kN	Error
1	1-1	50	51.06	2.12%	51.97	3.94%
	1-2	100	100.38	0.38%	102.15	2.15%
2	2-1	100	104.53	4.53%	103.47	3.47%
	2-2	200	211.85	5.93%	205.52	2.76%
	2-3	400	416.3	4.08%	379.88	5.03%
3	3-1	200	214.44	7.22%	203.92	1.96%
	3-2	400	382.35	4.41%	381.24	4.69%
	3-3	500	480.25	3.95%	492.82	1.44%
4	4-1	50	49	2%	48.2	3.61%

**Table 6:** Experimental scheme of harmonic force and the comparisons of *F*_m_, *F*_c_ and *F*_a_.

No.	Static Pre-load/kN	*F*_a_ (Harmonic Force)		*F*_m_ (Before Correction)		*F*_c_ (After Correction)	
		Amplitude/kN	Frequency/Hz	Amplitude/kN	Error	Amplitude/kN	Error
H1-1	100	20	2	14.87	25.65%	19.44	2.8%
H1-2	100	20	3	16.87	15.65%	19.35	3.25%
H2-1	100	40	2	31.23	21.93%	40.83	2.08%
H2-2	100	40	3	34.23	14.43%	39.25	1.9%

## References

[1] Romeo R.W., Floris M., Veneri F. (2006). Area-scale landslide hazard and risk assessment. Environ. Geol..

[2] Kamai T. (1998). Monitoring the process of ground failure in repeated landslides and associated stability assessments. Environ. Geol..

[3] Xu L.K., Li S.H., Liu X.Y., Feng C. (2007). Application of real-time telemetry technology to landslide in Tianchi Fengjie of Three Gorges reservoir region. Chin. J. Rock. Mech. Eng..

[4] Zan L., Latini G., Piscina E., Polloni G., Baldelli P. Landslides early warning monitoring system. Geoscience and Remote Sensing Symposium. Proceedings of the IEEE International Geoscience and Remote Sensing Symposium.

[5] Reeves B.A., Stickley G.F., Noon D.A., Longstaff I.D. Developments in monitoring mine slope stability using radar interferometry. Geoscience and Remote Sensing Symposium. Proceedings of the IEEE 2000 International Geoscience and Remote Sensing Symposium. Taking the Pulse of the Planet: The Role of Remote Sensing in Managing the Environment.

[6] Puglisi G., Bonaccorso A., Mattia M., Aloisi M., Bonforte A., Campisi O., Cantarero M., Falzone G., Puglisi B., Rossi M. (2005). New integrated geodetic monitoring system at Stromboli volcano (Italy). Eng. Geol..

[7] Zhang Y., Li H., Sheng Q., Wu K., Chen G. (2011). Real time remote monitoring and pre-warning system for Highway landslide in mountain area. J. Environ. Sci.-China.

[8] Wu K., Sheng Q., Zhang Y.H., Li Z.Y., Li H.X., Yue Z.P. (2010). Development of real-time remote monitoring and forecasting system for geological disasters at subgrade slopes of mountainous highways and its application. Rock. Soil. Mech..

[9] Perski Z., Hanssen R., Wojcik A., Wojciechowski T. (2009). InSAR analyses of terrain deformation near the Wieliczka Salt Mine, Poland. Eng. Geol..

[10] Jia G., Tian Y., Liu Y., Zhang Y. (2008). A static and dynamic factors-coupled forecasting model of regional rainfall-induced landslides: A case study of Shenzhen. Sci. China Technol. Sci..

[11] Xiang Y., Wang L., Wang Z.J., Yuan H., Guan Y.J. (2012). Key techniques for evaluation of safety monitoring sensors in water conservancy and hydropower engineering. Water. Sci. Eng..

[12] Cochrane C., Koncar V., Lewandowski M., Dufour C. (2007). Design and development of a flexible strain sensor for textile structures based on a conductive polymer composite. Sensors.

[13] Kuhinek D., Zoric I. Enhanced Vibrating Wire Strain Sensor. Proceedings of the 2007 IEEE Instrumentation and Measurement Technology Conference.

[14] Bourquin F., Joly M. (2004). A magnet-based vibrating wire sensor: design and simulation. Smart. Mater. Struct..

[15] Wang Q., Jiang J., Sun Y., Qi Y., Zhang J., Yin F., Li Z. (2012). Research and development on high performance anchor cable dynamometric system based on vibrating-wire sensor technology. Chin. J. Rock. Mech. Eng..

[16] Agioutantis Z., Kaklis K., Mavrigiannakis S., Verigakis M., Vallianatos F., Saltas V. (2016). Potential of acoustic emissions from three point bending tests as rock failure precursors. Int. J. Min. Sci. Technol..

[17] Karayannis C.G., Chalioris C.E., Angeli G.M., Papadopoulos N.A., Favvata M.J., Providakis C.P. (2016). Experimental damage evaluation of reinforced concrete steel bars using piezoelectric sensors. Constr. Build. Mater..

[18] Yan W., Chen W. (2006). Electro-mechanical response of functionally graded beams with imperfectly integrated surface piezoelectric layers. Sci. China Phys. Mech..

[19] Yang C., Lu Z. (2016). An interval effective independence method for optimal sensor placement based on non-probabilistic approach. Sci. China Technol. Sci..

[20] Yang C., Hou X., Wang L., Zhang X. (2016). Applications of different criteria in structural damage identification based on natural frequency and static displacement. Sci. China Technol. Sci..

[21] Gu H., Moslehy Y., Sanders D., Song G., Mo Y.L. (2010). Multi-functional smart aggregate-based structural health monitoring of circular reinforced concrete columns subjected to seismic excitations. Smart. Mater. Struct..

[22] Chalioris C.E., Papadopoulos N.A., Angeli G.M., Karayannis C.G., Liolios A.A., Providakis C.P. (2015). Damage evaluation in shear-critical reinforced concrete beam using piezoelectric transducers as smart aggregates. Open. Eng..

[23] Voutetaki M.E., Papadopoulos N.A., Angeli G.M., Providakis C.P. (2016). Investigation of a new experimental method for damage assessment of RC beams failing in shear using piezoelectric transducers. Eng. Struct..

[24] He M.C. (2009). Real-time remote monitoring and forecasting system for geological disasters of landslides and its engineering application. Chin. J. Rock. Mech. Eng..

[25] He M.C., Tao Z.G., Zhang B. (2009). Application of remote monitoring technology in landslides in the Luoshan mining area. Int. J. Min. Sci. Technol..

[26] He M., Gong W., Wang J., Qi P., Tao Z., Du S., Peng Y. (2014). Development of a novel energy-absorbing bolt with extraordinarily large elongation and constant resistance. Int. J. Rock. Mech. Min. Sci..

[27] Tao Z.G., Li H.P., Sun G.L. (2015). Development of monitoring and early warning system for landslides based on constant resistance and large deformation anchor cable and its application. Rock. Soil. Mech..

[28] Yang X., Hou D., Tao Z., Peng Y., Shi H. (2015). Stability and remote real-time monitoring of the slope slide body in the Luoshan mining area. Int. J. Min. Sci. Technol..

[29] Zhu Y.P., Kong D.R., Wang F. (2005). Sensors Principles and Applications.

[30] Chen J.P., Cheng W., Li M. (2016). Low-frequency compensation method of piezoelectric force sensor and experimental verification. J. Vib. Meas. Diag..

[31] Chen J.P., Cheng W. (2016). Low-frequency compensation of piezoelectric micro-vibration test platform. Tech. Vjesn..

